# Can we put a simplified algorithm for reconstruction of large scalp defects following tumor resection?

**DOI:** 10.1186/1477-7819-9-129

**Published:** 2011-10-15

**Authors:** Adel Denewer, Ashraf Khater, Omar Farouk, Mohammad Hegazy, Mahmoud Mosbah, Mohammad Hafez, Fayez Shahatto, Sameh Roshdy, Waleed Elnahas, Mohammad Kasem

**Affiliations:** 1 Department of Surgical Oncology, Oncology Center - Mansoura University (OCMU), Mansoura, Egypt; 2 Department of Neurosurgery, Mansoura University, Mansoura, Egypt

**Keywords:** Scalp reconstruction, large defect, scalp

## Abstract

**Background:**

Reconstruction of large scalp defects after tumor resection is a challenging problem. We aimed at putting an algorithm for reconstruction of those defects.

**Methods:**

Forty-two patients with scalp malignancies were enrolled in this study. Tumors were resected to a 1 cm negative margin and defects were reconstructed according to their size and to patient general condition.

**Results:**

No peri-operative mortality was encountered. Usage of free flaps was superior in cosmoses and function with an acceptable rate of complications.

**Conclusion:**

for scalp defects wider than100 cm^2^, the best tool of reconstruction is free flaps. Pedicled distant flaps are reserved if free flaps are not feasible or failed. Split thickness skin grafts are cosmetically inferior and not suitable for recurrent and irradiated tumours and better reserved for patients who cannot tolerate major operations.

## Background

Scalp reconstruction for large defects following tumor resection is a challenging complex problem for many reasons including the rather excessive width of these defects which commonly exceeds 50 cm^2^; the relatively limited elasticity of scalp tissues; the relatively poor vascularity (tumors are commonly locally advanced, recurrent after previous resection, or subjected to previous irradiation); and the remoteness of the scalp from donor sites. In addition, tumor resection may be a complex procedure when there is clavarial bony involvement that requires the attendance of a neurosurgeon. Another issue is the cosmetic appearance which mandates the use of hairy tissue. Associated co-morbid conditions especially cardiac disorders among elderly patients, who constitute a large category of cases with scalp tumors, represent another major challenge since they limit anesthetic tolerance and the use of distant flaps [1-3].

Reconstruction can be either immediate after obtaining free margins by frozen section or delayed after waiting for the results of paraffin section [4]. There are many options for the reconstruction of large defects. Primary closure either immediate or delayed remains the simplest option with hairy coverage; however it is not easy in cases with large defects which are more than 50 cm^2 ^[5]. The use of tissue expansion allows a larger area of coverage with satisfactory results, however, it has limitations for size coverage and it is not free of morbidities [6].

Local scalp flaps could be used successfully especially with extensive undermining. This option has the advantages of little morbidity and hairy coverage. Yet it is not effective in cases of large defects especially if they are larger than100 cm^2 ^[7].

A free split thickness skin graft can be used as a simple coverage for wide defects larger than 100 cm^2^, even if there is not sufficient periosteum through simply drilling down to the diploic layer of bone to improve the granulation process and to allow grafting later on. This graft can be done in the case of patients who cannot tolerate long anesthesia or those with associated cardiac co-morbidity but it has the disadvantage of inferior cosmetic appearance, less durability with possible trophic sores and the possibility of partial or total loss especially with prior irradiation and bad vascularity of the recipient site [8].

The use of distant pedicled flaps such as trapezius, pectoralis or latissimus dorsi flaps could be another solution which has the advantage of durable and efficient coverage of a wider area by means of a relatively simple technique. But it has the disadvantage of donor site morbidities and the increased possibility of flap ischemia which may lead to flap loss-especially with associated senility, diabetes, or co-morbid cardiac conditions. Moreover the flap is always bulky and with no hairy cover [9].

The last step in the reconstruction ladder is the use of free vascularized tissue transfer (free flaps) especially in the cases of wide defects associated with bad vascularity of the recipient site. Although their use has longer operative time, more anesthetic exposure, a greater possibility of donor and recipient sites morbidities with bulky non hairy coverage [10-12], they have the best cosmetic and functional outcome [13]

The aim of this study was to compare the different options of scalp reconstruction in terms of coverage scale, success rate, operative time, hospital stay, post-operative complications to produce a more simplified algorithm for choice of the proper type of reconstruction for each individual case.

## Methods

Forty-two patients with malignant tumors of the scalp were selected for this study. Twenty-four patients were males and eighteen were females. In all cases the tumors were primary except for four cases in which the patients had recurrent tumors after successful surgical resection (with 1 cm negative margin): three patients received pre-operative radiotherapy because of being locally advanced (clavarial bony involvement) with partial size regression (around 60%). Patients' demographic data are shown in table 1.

**Table 1:** Patient demographic data

	number	%
Total number:	42	100%
Gender:		
Male	24	57.14
Female	18	42.86
Mean age in years(range):	60.17 (45-77)	
Associated factors:		
Prior scalp surgery	4	9.52
Prior steroid therapy	0	0
Diabetes	10	23.81
Smoking history	15	35.71

The preoperative pathology was squamous cell carcinoma in 36 patients, Basal cell carcinoma in two patients and different pathology in four patients (3 cases with melanoma and one case with sebaceous adenocarcinoma). Tumor characteristics are shown in table 2.

**Table 2:** Tumor characteristics

	number	%
Tumor pathology:		
SCC	36	85.71
BCC	2	4.76
Melanoma	3	7.15
Sebaceous adenocarcinoma	1	2.38
Tumor location in the scalp:		
Central	3	7.14
Lateral	22	52.38
Frontal	5	11.91
Occipital	12	28.57
Primary versus recurrent:		
Primary	38	90.48
Recurrent:	4	9.52
Pre-operative radiation	3	7.14

The choice of the reconstruction procedure was determined according to the following criteria: a) Defect size which was classified into three groups as follows: > 50 cm^2 ^(large), > 100 cm^2 ^(very large) and > 200 cm^2 ^(extensive); b) the condition of the tumor bed (if there had been a history of prior irradiation or not and whether the condition of the granulation tissue was satisfactory or poor,... etc); c) local skin pliability (whether skin elasticity is satisfactory or poor); and d) patient anesthetic fitness and associated co-morbidities.

The reconstruction methods used in this study were as follows (table 3):

**Table 3:** Types of reconstruction and complications

	number	%
Reconstruction methods:		
Skin Graft	6	14.29
Rotational flaps	7	16.67
Distant pedicled flaps		
-Pectoral muscle flap	4	9.52
-Trapezius muscle flap	8	19.05
-Latissimus Dorsi flap	12	28.57
Free flap	5	11.90
Major complications:		
Total flap loss	1	2.38
Partial flap loss	4	9.52
Moderate hematoma	3	7.14
Wide wound dehiscence	3	7.14
pneumonia	1	2.38
Minor complications		
Minor graft or flap loss	10	23.81
Wound sepsis	3	7.14
Tumor recurrence within the follow up period:	1	2.38

1- Skin Graft was used with six patients who were all more than 70 years old, with no prior irradiation, defects were > 100 cm^2 ^with lack of skin pliability. Three patients in this group needed drilling of the outer table to enhance granulation tissue formation.

2- Rotation flaps were used with 7 patients whose defects were ranging from 50 cm^2 ^to100 cm^2 ^with satisfactory skin pliability and satisfactory anesthetic tolerance.

**Figure 1:**
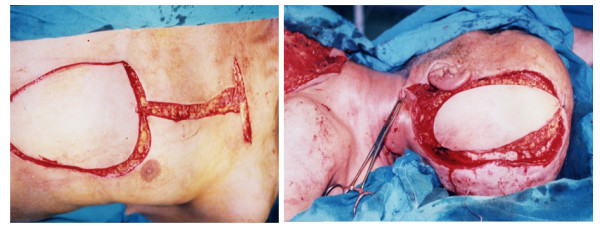
**large defect reconstructed with Pectoralis major myocautaneous flap**.

**Figure 2:**
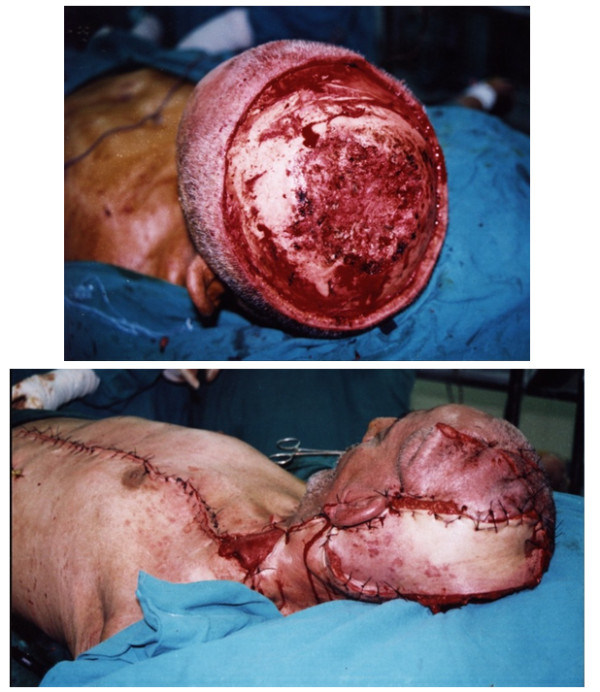
**very large defect closed by combined local flaps plus Pectoralis major myocautaneous flap**.

3- Distant pedicled flaps were used as follows: Pectoral muscle flap with 4 male patients (Figures 1&2); Trapezius muscle flap with 8 patients; and Latissimus dorsi flap with 12 patients. Patients in this group had defects which were > 100 cm^2 ^or less with lack of skin pliability or had already received pre-operative irradiation and had good anesthetic tolerance.

**Figure 3:**
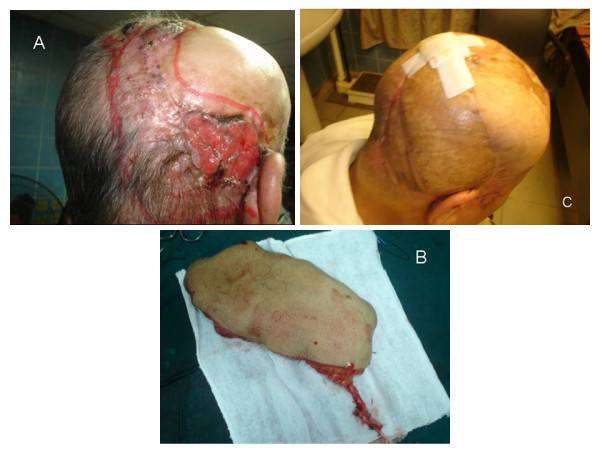
**A case reconstructed with antero-lateral thigh flap based on the lateral circumflex femoral artery**. A: preoperative view. B: the flap with its vascular supply, C: postoperative view.

4- Free tissue transfer: Free Latissimus dorsi flap was used with four patients and antero-lateral thigh flap with one patient (Figure 3). Defects were > 100 cm^2 ^with good anesthetic tolerance.

All patients underwent immediate reconstruction after ascertaining that that there is a 1 cm safety margin by frozen section. Neurosurgical attendance was required in six patients. Three of these patents had clavarial skull bony involvement while the other three patients had an infiltrated deep safety margin by frozen section. Excision of part of the outer table of the skull was done in three cases and full thickness excision was done in the remaining cases with no CSF leak.

Immediate and late follow up was undertaken for the evaluation of:

1-Viability of the cover method and the degree of success of coverage using postoperative duplex and C.T angiography

2-Recipient and donor sites morbidities

3- Operative time and hospital stay

4-Subjective cosmetic acceptance

5- Immediate and late overall morbidity and mortality

6-Tumor recurrence within the follow-up period using C.T.

## Results

The total number of patients in this study was forty-two. Twenty-four patients were males (57.14%) and 18 patients were females (42.86%) with a mean age of 60.17 years old (ranging from 45 years old to 77 years old). Thirty-eight patients had primary tumours (90.48%) while 4 had recurrent tumours (9.52%).Three patients received pre-operative irradiation (7.14%) (With clavarial bony involvement) and showed around 60% down staging. Thirty-six patients had squamous cell carcinoma (85.71%), two patients had Basal cell carcinoma (4.76%) and the other four patients had different pathology (3 patients with melanoma and one patient with sebaceous adenocarcinoma).

All cases underwent radical excision with 1 cm safety margin as proved by frozen section assessment. An infiltrated lateral margin was encountered in four cases and was treated by wider excision. In three cases there was infiltration of the deep margin that was treated by excision of part of the skull with the aid of a neurosurgical team. In all cases final paraffin section staining of margins was free of tumor. The reconstruction method was tailored for every patient according to the criteria specified earlier.

In general, there was no peri-operative mortality. Complications were classified into major (most of them were managed operatively) and minor (most of them were managed conservatively).

Major complications included (table 3):

1-Total flap loss: This was noticed in one patient with squamous cell carcinoma excised with a good safety margin and reconstructed using free Latissimus flap. Flap loss was due to venous thrombosis and it was manifested by the fifth post-operative day. Trial vascular refashioning was carried out but it failed and multiple skin grafts were tried with partial success. This patient was 62 years old, non-diabetic with no history of steroid intake or prior irradiation.

2-Partial flap loss: It was encountered in four patients; three of whom had a free Latissimus flap while one patient had a pedicled trapezius flap. All patients had primary tumors, were under 70 years old, with no history of steroid intake or prior irradiation but one case was diabetic five years ago and was on insulin treatment. The lost parts were treated by debridement and split thickness skin graft after granulation.

3-Moderate hematoma (about 200 cc in volume), that was treated by operative evacuation, was encountered in one patient of reconstruction using free Latissimus flap and two patients of pedicled Latissmus flap. All patients had primary tumors with no history of diabetes or irradiation but two of them were hypertensive under medical control.

4-Wide wound dehiscence (involving more than 60% of the wound) was encountered in three patients with pedicled Latissimus and was managed by delayed closure. The demographic data of those patients were; two cases with diabetes more than ten years under insulin control and one patient who received pre-operative radiotherapy.

5-Peri-operative systemic complications: One case with free Latissimus flap suffered from pneumonia and was treated medically with resultant prolonged hospital stay up to two weeks.

Minor complications included (table 3):

1-Minor graft or flap loss (less than 20%): This was noticed in ten patients four of whom had a pedicled Latissimus flap, three patients had skin grafts while three patients had local flaps. All patients passed smoothly with conservative dressing.

2-Wound sepsis was encountered in three patients: two had free Latissimus while one had a pedicled Trapezius flap and all were treated conservatively by simple drainage and antibiotic based on culture and sensitivity.

3-Donor site morbidity: Seroma formation was the only recorded donor site morbidity. It was recorded in six patients with a pedicled Latissimus flap and in two patients with a free Latissimus flap. It was managed conservatively.

The mean operative time was 21 minutes (rangeing from 15 to 25 minutes) in cases of skin grafts; 34.29 minutes (ranging from 30 to 40 minutes) in cases of local flaps;72.2 minutes (ranging from 64 to 84 minutes) in cases of pedicled flaps; and 162 minutes (ranging from 120 to 180 minutes) in cases of free flaps (table 4).

**Table 4:** Specific data of each method of reconstruction

	Skin grafts	Local flaps	Pedicled flaps	Free flaps
Age (year):				
Mean (range)	74.5 (72-77)	56.71 (45-65)	59.29 (47-70)	52 (45-60)
Defect size (cm):				
Mean (range)	146.67 (120-180)	75 (60-90)	144.58 (105-185)	177 (160-190)
Operative time (minutes):				
Mean (range)	21 (15-25)	34.29 (30-40)	72.2 (64-84)	162 (120-180)
Hospital stay (days):				
Mean (range)	3.67 (3-4)	5.86 (5-7)	7.96 (6-11)	11.4 (9-14)
Major complications:	0	0	6	5
Minor complications:	3	3	5	2

The mean hospital stay was 3.67 days (ranging from 3 to 4 days) in patients of skin grafts; 5.86 days (ranging from 5 to 7 days) in patients of local flaps; 7.96 days (ranging from 6 to 11 days) in patients of pedicled flaps; and 11.4 days (ranging from 9 to 14 days) in patients of free flaps (table 4).

A subjective score from one to ten to assess the cosmetic outcome as noticed by the patient was used. It was found that the highest scores were associated with the use of local and free flaps (mean 8, average 7-9), followed by pedicled flaps (mean 6, average 5-7). The lowest scores were associated with skin grafts (mean 4, range 3-5) (table 4).

During a follow up period of 24 months; one patient showed local recurrence with evidence of fixity to skull periosteum, which was treated by surgical resection of skull bone with the aid of the neurosurgical team followed by the use of a pedicled Latissimus flap.

## Discussion

Scalp reconstruction differs from reconstruction elsewhere in the difference of the anatomic nature of the scalp being with limited elasticity, which makes primary closure for wounds larger than 50 cm^2 ^somewhat difficult even with undermining [2].

The use of tissue expansion is an option to increase the possibility of primary closure but unfortunately this option was not suitable for most of our patients for many reasons: most of the defects were too large for this technique; many patients had a lack of skin pliability and some had recurrent tumors or received radiotherapy; in addition this technique is neither free of complications nor suitable for immediate coverage [6,14].

The use of local flaps has the advantage of coverage with hair bearing skin and of achieving an acceptable cosmetic outcome without need to disturb other areas. However this option is not easily applied in very large defects especially those with skin lacking pliability [2]. Although local flaps may be performed after extensive undermining to allow for closure of both the acquired and donor defects, it is preferable to be more cautious by creating a local flap large enough to cover the ablative defect in its entirety and to graft the donor site defect. Using this approach, complications can be minimized even in patients of large defects.

Skin grafting with a relatively thick split thickness graft is an option, which is suitable for any defect and is considered a relatively simple technique, but it has some disadvantages. In addition to its inferior cosmetic appearance especially when meshed, it is not so durable and is also liable for local ulceration. Although it could be used even with deficient periosteum through drilling of the outer table but in this situation the graft-take and durability are always inferior. Based on our experience, it is better to be avoided. The same is also noticed in patients with prior irradiation [2]. For this, we reserve grafting to cases in which flaps are not feasible or contraindicated.

Pedicled Pectoralis major and Latissimus dorsi flaps have relatively reliable vasculature and long arc of rotation. These flaps produce durable and reliable coverage with the least complications whereas their disadvantages are the relative bulk and non-hairy cover [9].

Although free flaps are somewhat time consuming and technically demanding because of the anesthetic burden they provide the best cosmetic and functional results [13]. Among the options available in the long list of free flaps, Latissimus dorsi and antero-lateral thigh flap are our choice because they have a reliable vascular supply and an easier harvest with the least donor site morbidity. We do not recommend omental flap which requires abdominal exploration and subsequent skin grafting [15].

Some centers recommend excision of skin and subcutaneous tissue with preservation of small skin island for monitoring of vascularity with coverage by skin graft aiming at the reduction of the unsightly large bulk [1]. In our center, however, we prefer immediate reconstruction based on the negativity of margins by frozen section. There were no positive margins in paraffin block in any of our patients. Some other centers do not rely on the results of frozen section and prefer delayed reconstruction after negative paraffin block [1,16]. However, in those centers patients who presented this diagnostic dilemma had angiosarcoma and melanocytic tumors and were among those receiving radiotherapy. In our study, most of cases had squamous and basal cell carcinoma [17].

**Figure 4:**
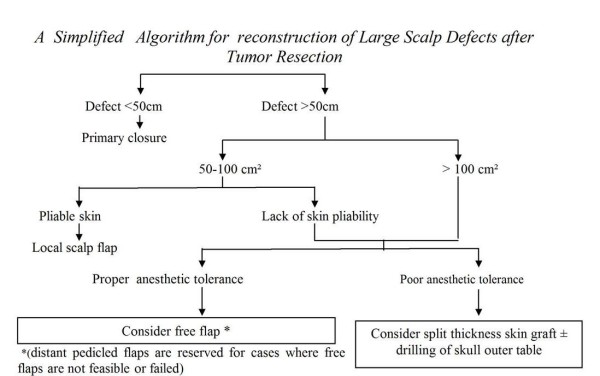
**A Simplified Algorithm for reconstruction of Large Scalp Defects after Tumor Resection**.

Finally, we put an algorithm for the reconstruction of scalp defects based on the defect size, state of the local skin and the patient general condition (Figure 4).

## Conclusion

Scalp reconstruction is an interdisciplinary work that mandates integration of oncosurgeons, neurosurgeons, anesthetists, and pathologists. Choice of method of reconstruction must follow the reconstruction ladder putting in mind certain factors as the defect size, skin pliability, local tissue vascularity, patient general condition and associated co morbidity. Our preference for reconstruction of huge scalp defects is the use of free flaps unless there is contraindication.

## Conflict of interests

The authors declare that they have no competing interests.

## Authors' contributions

AD carried out the surgical techniques, conceived of the study and drafted the manuscript. AK put the design of the study, drafted the manuscript and approved the final manuscript. OF participated in the design of the study and drafted the manuscript. MHe assisted in surgical techniques and drafted the manuscript. FS assisted in surgical technique. MM performed the statistical analysis, and participated in its coordination. MHa assisted in the statistical analysis, and participated in its coordination. SR assisted in surgical techniques. WE assisted in surgical techniques. MK assisted in surgical techniques. All authors read and approved the final manuscript.

## References

[B1] Kruse-Lösler B Presser D Meyer U Reconstruction of large defects on the scalp and forehead as an interdisciplinary challenge: Experience in the management of 39 cases EJSO 2006 32 1006 1014 16806795 10.1016/j.ejso.2006.05.001

[B2] Newman MI Hanasono MM Disa JJ Scalp reconstruction: A 15 year experience Annals of plastic surgery 2004 52 5 501 506 10.1097/01.sap.0000123346.58418.e6 15096938

[B3] Joannides C Fossion E McGrouther AD Reconstruction for large defects of the scalp and cranium J Craniomaxillofac Surg 1999 27 145 152 10.1016/S1010-5182(99)80042-0 10442304

[B4] Smith-Zagone MJ Schwartz MR Frozen section of skin specimens Arch Pathol Lab Med 2005 129 1536 43 16329726 10.5858/2005-129-1536-FSOSS

[B5] Raposio E Nordstrom RE Santi PL Undermining of the scalp: quantitative effects Plast Reconstr Surg 1998 101 1218 1222 10.1097/00006534-199804050-00007 9529204

[B6] Manders EK Schenden MJ Furrey JA Soft tissue expansion: concepts and complications Plast Reconstr Surg 1984 74 493 507 10.1097/00006534-198410000-00007 6484036

[B7] Kroll SS Margolis R Scalp flap rotation with primary donor site closure Ann Plast Surg 1993 30 452 5 10.1097/00000637-199305000-00011 8342932

[B8] Liu C Liao N Treatment of overtime avulsion of scalp with split thickness scalp skin grafting: 7 cases of reports Zhongguo Xiu Fu Chong Jian Wai Ke Za Zhi 2003 17 5 388 90 Chinese 14551937

[B9] Maxwell GP Leonard LG Manson PN Hoopes JE Craniofacial coverage using the latissimus dorsi myocutaneous island flap Ann Plast Surg 1980 4 410 21 10.1097/00000637-198005000-00009 7436271

[B10] Hussussian CJ Reece GP Microsurgical scalp reconstruction in the patient with cancer Ann Plast Surg 2002 109 1828 1834 10.1097/00006534-200205000-00008 11994580

[B11] McCombe D Donato R Hofer SO Free flaps in the treatment of locally advanced malignancy of the scalp and forehead Ann Plast Surg 2002 48 600 606 10.1097/00000637-200206000-00006 12055428

[B12] Furnas H Lineaweaver WC Alpert BS Buncke HJ Scalp reconstruction by microvascular free tissue transfer Ann Plast Surg 1990 24 431 44 10.1097/00000637-199005000-00007 2350153

[B13] Van Driel AA Mureau MA Goldstein DP Gilbert RW Irish JC Gullane PJ Neligan PC Hoer SO Aesthetic and oncologic outcome after microsurgical reconstruction of complex scalp and forehead defects after malignant tumor resection: an algorithm for treatment Plast Reconstr Surg 2010 126 2 460 70 10.1097/PRS.0b013e3181de2260 20679830

[B14] Gürlek A Alaybeyoğlu N Demir CY Aydoğan H Aesthetic reconstruction of large scalp defects by sequential tissue expansion without interval Aesthetic Plast Surg 2004 28 245 50 10.1007/s00266-004-4008-3 15599540

[B15] Losken A Carlson GW Cullbertson JH Scott Omental free flap reconstruction in complex head and neck deformities Head Neck 2002 24 326 331 10.1002/hed.10082 11933173

[B16] Manstein ME Manstein CH Smith R How accurate is frozen section for skin cancers? Ann Plast Surg 2003 50 6 607 9 10.1097/01.SAP.0000069073.38391.91 12783011

[B17] Prieto VG Argenyi ZB Barnhill RL Are en face frozen sections accurate for diagnosing margin status in melanocytic lesions? Am J Clin Pathol 2003 120 203 8 10.1309/J1Q0V35EUTMVR193 12931550

